# Lack of association between *SREBF-1c* gene polymorphisms and risk of non-alcoholic fatty liver disease in a Chinese Han population

**DOI:** 10.1038/srep32110

**Published:** 2016-08-30

**Authors:** Xian-E. Peng, Feng-Lin Chen, Wenjuan Liu, ZhiJian Hu, Xu Lin

**Affiliations:** 1 Department of Epidemiology and Health Statistics, the Key Laboratory of Environment and Health, universities and colleges in Fujian, School of Public Health, Fujian Medical University, 1 Xueyuan Road, Minhou, Fuzhou 350108, China; 2 Key Laboratory of Ministry of Education for Gastrointestinal Cancer, Fujian Medical University, 1 Xueyuan Road, Minhou, Fuzhou 350108, China; 3 Department of Gastroenterology, Union Hospital of Fujian Medical University, 29 Xinquan Road, Fuzhou 35001, China; 4 Fujian Key Laboratory of Tumor Microbiology, Fujian Medical University, 1 Xueyuan Road, Minhou, Fuzhou 350108, China

## Abstract

The transcription factor sterol regulatory element-binding protein-1c (*SREBP-1c*) is a key regulator of lipogenesis and insulin sensitivity, and is associated with non-alcoholic fatty liver disease (NAFLD). Here, we assessed the impact of common single nucleotide polymorphisms (SNPs) in *SREBF-1c* on NAFLD susceptibility and associated metabolic phenotypes in a Han Chinese population. Four common SNPs (rs62064119, rs2297508, rs11868035 and rs13306741) in the *SREBP-1c* gene were selected and genotyped in 593 patients with NAFLD and 593 healthy controls. Unconditional logistic regression was performed to assess the risk of NAFLD by determining odds ratios and 95% confidence intervals (CIs). No significant differences in genotype and allele frequencies of these four SNPs were found between the NAFLD population and the controls (all *P* > 0.05). In addition, we did not find any association between the *SREBF-1c* SNPs and the clinical and biochemical parameters, such as body mass index, total cholesterol, high density lipoprotein-and low density lipoprotein-cholesterol or systolic and diastolic blood pressure, except that the rs2297508 C-allele or rs11868035 G-allele showed significant associations with lower triglyceride levels in control subjects (P < 0.01). Our findings suggested that the four polymorphisms in *SREBF-1c* gene are not associated with risk of NAFLD in the Chinese Han population.

Sterol regulatory element-binding proteins (SREBPs) are a family of transcription factors that activate the entire program of cholesterol and fatty acid synthesis in the liver^1^,^2^. To date, three SREBP isoforms, i.e., SREBP-1a, -1c, and -2, have been identified and characterized. SREBP-1a and SREBP-1c are encoded by a single gene named sterol regulatory element–binding protein gene (*SREBF*)-1 (via specific promoters and alternative splicing)^3^,^4^, and SREBP-2 is encoded by a separate gene, *SREBF-2*^5^.

SREBP-1c is the main SREBF1 isoform, which is highly expressed in many tissues in mice and humans, including the liver, adipose tissue and skeletal muscle^6^, and can enhance transcription of genes involved in fatty acid and triacylglycerol synthesis^7^,^8^. Overexpression of SREBP-1c induced insulin resistance, diabetes, non-alcoholic fatty liver disease (NAFLD), and accelerated atherosclerosis in mice^9^,^10^,^11^,^12^. Given the mediating role of SREBF-1c in the transcription of genes involved in de novo lipogenesis and triglyceride-rich lipoprotein metabolism, the *SREBF-1c* gene could be considered as a good candidate indicator to predict metabolic syndromes disorders.

NAFLD is a spectrum of disorders ranging from simple fatty liver (NAFL) to non-alcoholic steatohepatitis (NASH), with or without fibrosis/cirrhosis^13^. The mechanism leading to NAFLD remains unclear; however, it is now largely accepted that the risk of NAFLD increases exponentially with the presence of metabolic syndrome or its components, including central abdominal obesity, hypertension, dyslipidaemia and type 2 diabetes (T2D)^14^,^15^. Therefore, it is well recognized that NAFLD is the hepatic manifestation of metabolic syndrome^16^. Single nucleotide polymorphisms (SNPs) within the *SREBF-1c* gene have been connected to metabolic syndrome disorders in humans^17^,^18^. Specifically, the SNP rs11868035 A/G, located in the intron of the *SREBF-1c* gene modulates the risk of type 2 diabetes, insulin resistance, obesity and hypercholesterolemia in different ethnicities^19^,^20^,^21^. In addition, a nearby-located synonymous SNP in exon 18 (rs2297508), located 216 bp downstream of rs11868035 A/G, was also found to be associated with obesity, diabetes and male-specific hypertriacylglycerolaemia in a French population^22^. Although the effects of *SREBF-1c* gene variants on metabolic syndromes are well documented, little is known about their effects on NAFLD. To date, only one study has reported that rs11868035 A allele conferred increased risk of severe steatosis and non-alcoholic steatohepatitis in a North-Western Italy population^19^.

Given the strong association between NAFLD and multiple metabolic diseases, the aim of this study was to identify whether polymorphisms in the *SREBF-1c* gene are associated with increased risk of NAFLD in a population from China. Subsequently, we also examined the association of each SNP with the metabolic phenotypes.

## Results

### Descriptive characteristics of the study population

The descriptive characteristics of the study participants according to NAFLD status (NAFLD patients *vs*. controls) are shown in Table 1. Briefly, there were no statistically significant differences between the NAFLD patients and control subjects in terms of age, sex, marital status, education, smoking or income status (all P > 0.05, see), indicating that the frequency matching was adequate. However, the NAFLD patients had a lower percentage of tea drinkers, as well as higher occurrence of most of the risk factors for the metabolic syndrome, including elevated blood pressure, body mass index (BMI), fasting plasma glucose (FPG), total cholesterol (TC), trigylcerides (TGs), low density lipoprotein (LDL)-C and decreased high density lipoprotein (HDL)-C (each P < 0.05), whereas the opposite was observed for controls. Additionally, levels of alanine transaminase (ALT) and aspartate transaminase (AST) were significantly higher in the patients with NAFLD than in the controls (both P < 0.05).

### Association between individual genotypes and risk of NAFLD

SNPs information for the *SREBF-1c* gene is shown in Table 2. These SNPs were genotyped in all 1,186 individuals using iPlex technology based on a MassARRAY platform, and were commonly distributed in the study samples. The corresponding frequencies of the rs62064119 G, rs2297508 C, rs11868035 G and rs13306741 A alleles among the participants were 0.560, 0.130, 0.133 and 0.047 in NAFLD patients, and 0.556, 0.120, 0.121 and 0.044 in controls, respectively. In the univariate analysis, after multiple comparison correction by permutation tests, there were no significant differences in the allele frequency of these four SNPs between the control group and NAFLD patients (empirical *P-*values = 0.39–0.87).

Distributions of the genotypes of these four polymorphisms in NAFLD patients and control subjects and their associations with NAFLD risk are summarized in Table 3. All of the observed genotype frequencies conformed to the Hardy–Weinberg equilibrium (HWE), both in NAFLD patients and the controls (*P*_NAFLD_ = 0.130–0.054; *P*_control_ = 0.350–0.882, respectively). There were no significant differences in the genotype distribution between the two groups (*P* > 0.05). Variables such as age, sex, income, marital status, education, smoking, tea drinking, BMI and other clinical features might affect the development of NAFLD; therefore, unconditional logistic regression analysis was further used to estimate associations between the *SREBF-1c* genotypes and the risk of NAFLD. After controlling for the effect of these confounding factors, we also did not find any significant effects of *SREBF-1c* SNPs on the risk of NAFLD.

We further investigated the effects of *SREBF1c* variants on liver function tests. As shown in Table 4, both the A-allele of rs11868035 and G-allele of rs2297508 were associated with significantly increased ALT levels (*P* < 0.05) in all studied subjects (i.e. n = 1186, without separating them into controls and cases). However, no statistical significance for the effect of *SREBF-1c* gene variants on liver function was found in both NAFLD and control subjects (each *P* > 0.05).

The linkage disequilibrium (LD) pattern among *SREBF-1c* polymorphisms was further evaluated in our study population. The disequilibrium statistics between rs62064119, rs2297508, rs11868035 and rs13306741 showed a very strong LD (D′ = 0.90–0.99) with each other and a haplotype block including rs2297508 and rs11868035 was detected. As shown in Table 5, we did not detect any significant difference in the overall distribution of haplotypes between cases and controls (*P*_sim for global score test_ = 0.29).

### Association of Clinical Parameters with different *SREBF-1c* gene variants in NAFLD Patients and controls

Given that *SREBF-1c* gene variants have been reported to be associated with the components of metabolic syndrome and the high association between NAFLD and multiple metabolic diseases, we further investigated whether *SREBF-1c* variants might be associated with alterations in metabolic parameters related to NAFLD in our population. We assessed the relationship between the four *SREBF-1c* variants and FPG, BMI and plasma lipids in the NAFLD and control subjects, respectively. As shown in Tables S1–4, no statistical significance for the associations between the *SREBF-1c* gene variants and all selected clinical parameters was found in both NAFLD and control subjects (each *P* > 0.05), except that the rs2297508 C-allele or rs11868035 G-allele showed significant associations with lower TG level in control subjects (rs2297508 GG *vs*. GC + CC: 1.27 ± 0.69 *vs*. 1.08 ± 0.44, p = 0.003, rs11868035 AA *vs*. AG + GG: 1.26 ± 0.68 *vs*. 1.08 ± 0.45, p = 0.004).

## Discussion

In this study, to evaluate whether SNPs in the whole *SREBF-1c* gene are associated with NAFLD or metabolic parameters related to this disease, we enrolled a total of 1186 unrelated Chinese adults in a case-control study to investigate the relationship between four *SREBF-1c* polymorphisms (rs62064119, rs2297508, rs11868035 and rs13306741) and the risk of NAFLD. Our results failed to reveal an association between SNPs or common haplotypes of *SREBF-1c* and NAFLD in the Chinese Han population. In addition, we did not find any association between the *SREBF-1c* SNPs and BMI, TC, HDL-and LDL-cholesterol or systolic and diastolic blood pressure, both in NAFLD and control subjects, except that the rs2297508 C-allele or rs11868035 G-allele showed significant associations with a lower TG level in control subjects. Thus, our results suggested that these four SNPs in *SREBF-1c* gene are not associated with the risk of NAFLD in the Chinese Han population.

SREBP-1c is a well-known regulator of hepatic lipogenesis and insulin sensitivity. Animal studies have shown that increased hepatic SREBP-1c is associated with insulin resistance, diabetes and hepatic steatosis, partially by the enhanced synthesis of lipotoxic saturated fatty acids and increased hepatic gluconeogenesis^23^,^24^. Two *SREBP-1c* SNPs (rs11868035 A/G and rs2297508 G/C) were evaluated previously in relation to metabolic diseases risk, and carriers of the rs11868035 A allele and rs2297508 G allele have been reported to significantly increase the risk of type 2 diabetes^18^,^21^. However, little is known about the effect of *SREBF-1c* gene variants on NAFLD, except that Musso *et al*. investigated the relationship between *SREBF-1c* rs11868035 A/G polymorphism and risk of NAFLD, and found that rs11868035 A allele conferred increased risk of severe steatosis and non-alcoholic steatohepatitis in a North-Western Italy population^19^. This association, however, was not observed in our study: we did not observe any significant difference in rs11868035 genotypes between NAFLD cases and controls. Moreover, no association with the other three SNPs or any common haplotypes of the *SREBPF-1c* gene and NAFLD risk was observed. Since lack of association can always be due to lack of power, a small effect on NAFLD cannot be ruled out. However, based on our sample size and the minor allele frequency, the power of detecting an *OR* of 1.2 at a two-sided α = 0.05, were >80% respectively. These results suggest that these four SNPs in the *SREBF-1c* gene may not influence fatty liver disease in our population. The inconsistency may be explained by the following reasons: Firstly, the most prevalent alleles of rs11868035 and rs2297508 are different for various populations of different genetic background^18^,^19^,^25^. Genome diversity data extracted from http://www.ncbi.nlm.nih.gov/SNP/ shows that the frequencies of rs2297508 G allele and rs11868035 A allele in Chinese population are much higher than those in CEU population (rs2297508 G allele: 82.2% vs 25.8%; rs11868035 A: 83.7% vs 28.1%), which may partially explained the inconsistency. Secondly, it is now largely accepted that NAFLD is multifactorial, and various genetic alterations and environmental factors influence the development and progression of NAFLD^26^,^27^. A genome-wide association study (GWAS) showed that the rs738409 G allele of Padiponutrin/patatin-like phospholipase domain-containing protein 3 (*PNPLA3*) was significantly associated with increased risk of NAFLD^28^, which was further replicated in several populations^29^,^30^. Moreover, Krawczyk *et al*. reported that carriers of both *PNPLA3* rs738409 and *SREBP1c* rs2297508 risk genotypes significantly promoted hepatic fibrogenesis, as compared to patients carrying none of these variants^31^. Silico analysis also revealed the presence of a sterol response element (SRE) binding site on the *PNPLA3* gene promoter and *PNPLA3* expression is induced directly by *SREBP1c*^32^. In fact, we previously demonstrated that the rs738409 GG genotype of *PNPLA3* significantly increased the risk of NAFLD in our cohort (adjusted OR = 2.25, 95% *CI*: 1.46–3.45 for GG genotype vs CC genotype; *P* = 0.001)^30^.However, in the present study, we did not find any joined effects between *PNPLA3* rs738409 and *SREBP1*c polymorphisms on NAFLD (data not shown). Finally, the inconsistent results may be partially explained by the differences in the study design or statistical power. Thus, further studies in large population-based cohorts from different ethnicities are warranted to identify the role of *SREBP1-c* polymorphisms in NAFLD.

We further investigated the effects of *SREBF1c* variants on liver function tests (LFTs) and the results showed that both the A-allele of rs11868035 and G-allele of rs2297508 were associated with significantly increased ALT levels (*P* < 0.05) in the entire cohort (i.e. n = 1186, without separating them into controls and cases), but not in separate analysis for cases and controls. The explanation for this inconsistency was that, we conducted a case-control study of which the subjects were recruited based on their disease status, therefore the entire subjects (including cases and control) were not a random sampling from source population, and the association between *SREBF1c* variants and increased LFTs in the entire subjects may be spurious due to selection bias. Up to date, there was no report about the association between *SREBF-1c* variants and LFTs, further studies in a large random sampling are warranted to identify the role of *SREBP1-c* polymorphisms in LFTs.

Previous reports regarding associations between *SREBF-1c* variants and quantitative metabolism have been inconclusive. Previous studies have indicated an association of the rs11868035 variant with increased fasting total, and LDL, cholesterol levels in Caucasian and Danish populations, respectively^21^,^22^. In contrast, studies from Austrian and French populations did not show any association of the rs2297508 variant with fasting cholesterol level^33^,^34^ In the present study, we also evaluated the influence of *SREBF-1c* SNPs on metabolic syndrome component traits and the results showed that the rs2297508 GC + CC genotypes or rs11868035 TC + CC genotypes showed significant associations with lower TG levels compared with wild-type genotypes in the control group, which suggests that these variant have stronger effects on some metabolic traits than on NAFLD. Rs2297508 is a synonymous mutation, and rs11868035 is intronic SNP. Although these two SNPs associated with serum TG do not directly change protein structure, they could influence various aspects of mRNA metabolism^35^. Further functional studies should provide additional insights into the role of these variants in lipid metabolism.

Our study had strengths and limitations. The strengths of our study include our ability to match cases of NAFLD with controls to enable us to find variants that might be associated with NAFLD. Secondly, the moderate sample size employed in this study could have reduced type II errors. Finally, extensive information on anthropometrics and lifestyle factors were collected in this study to adjust for confounding factors. However, the potential limitations of the present study should also be considered. Firstly, there was a recruitment bias resulting from the retrospective case–control study design that could be a possible confounding factor in our results; however, the results did not appear to be seriously affected by this bias, because the control group was in HWE. Secondly, the ultrasonographic definition of steatosis may have missed some cases of milder steatosis. However, recent standardized criteria have significantly improved the ultrasonographic diagnostic accuracy to detect even minor degrees of steatosis^36^. Finally, our study was performed on a specific population (i.e., a health examination population of Han Chinese) and thus might not be applicable to the entire population or other ethnicities. Further population-based association studies are warranted to verify our results, especially those including different ethnic groups.

In summary, our study confirmed that the polymorphisms of *SREBF-1c* gene are not related to the risk of NAFLD in the Chinese Han population. However, other genetic risk factors for this disease may exist. Further research is needed to screen novel biomarkers and explore the mechanism underlying the pathogenesis of NAFLD.

## Materials and Methods

### Study population

A 1:1 frequency-matched case–control Study of men and women aged 20–78 years was performed in a health examination centre of the Union Hospital of Fujian Medical University, from June of 2008 to May of 2012. Participants with anyone status of the following were excluded: 1) Alcohol abuse in the past year (weekly ethanol consumption ≥140 grams for men; ≥70 grams for women); 2) hepatitis B or hepatitis C virus infection; and 3) had received lipid-lowering treatment or any other drug-modifying lipid measures. The study design has been previously described in detail elsewhere^37^. Briefly, the case–control study comprised 593 NAFLD cases and 593 controls, each matched in terms of age (within 5 years), sex, ethnicity, occupation, geographical location and recruitment date. A case of NAFLD was defined by an abdominal ultrasonography made by two experienced radiologists blinded to the participants’ data. An ultrasonographic diagnosis of steatosis was based on recently proposed standardized criteria, which have substantially improved the diagnostic accuracy of ultrasonography^36^. Patients with secondary causes of steatosis, including total parenteral nutrition and the use of drugs known to precipitate steatosis were excluded. In addition, patients with any of the following diseases were excluded from participation in this study: autoimmune hepatitis, drug-induced liver disease, primary biliary cirrhosis and primary sclerosing cholangitis. Healthy individuals without steatosis by abdomen ultrasonography (n = 593) were recruited randomly from the same population during the same study period: these subjects underwent a routine health check and were free of any known major diseases. All enrolled subjects were of Chinese Han ethnicity. Informed, written consent was obtained from all participants. The studies were conducted in accordance with the Declaration of Helsinki II and were approved by the local ethics committees of the Union Hospital of Fujian Medical University and Fujian Medical University.

### Demographic and clinical data

All subjects underwent a complete physical examination in the morning after a 12-hour overnight fast and 48-hour alcohol abstinence. Health examination included anthropometric measurements, abdominal ultrasonography, a questionnaire on health-related behaviour (smoking, and tea drinking), and biochemical determinations. Smokers were defined as subjects who had smoked at least 100 cigarettes during their lifetime and were classified as smokers *vs*, non-smokers. Tea drinkers were defined as individuals who drank at least one cup of green tea per day for more than half a year and classified as drinkers *vs*, non-drinkers. Weight and standing height were measured in a standardized fashion by a trained examiner. The BMI was calculated as the ratio of weight (kilograms) to the square of height (meters) (weight (kg) divided by height squared (m^2^)). Overweight and/or obesity were defined as having a BMI ≥25 kg/m^2^. Three resting blood pressure readings were obtained at 1-min intervals; the second and third systolic and diastolic pressure readings were averaged and used for analysis. After an overnight fast, venous blood samples were collected from an antecubital vein into vacutainer tubes containing EDTA. Plasma was separated by centrifugation (2,500 × *g* for 10 min at 4 °C), and samples were aliquoted and frozen for subsequent analyses. The levels of TC, HDL-C and LDL-C, TGs, FPG), and uric acid (UA), in addition to liver function tests, were measured by standard clinical laboratory techniques, which have been described previously^37^.

### SNP selection and genotyping

The genomic region harbouring *SREBF-1c* was examined to select SNPs based on the LD patterns of Chinese Han population in Beijing (CHB) from the International HapMap Project SNP database [HapMap Data Rel. 28 Phase II + III, August 10, on National Center for Biotechnology Information (NCBI) B36 assembly, dbSNP b126, accessed April 2013]. We then used the pairwise tagging method of the Haploview v4.2 software (Broad institute, Cambridge, MA, USA) to capture SNPs with a minimum minor allele frequency (MAF) of > 0.02 and a minimum r^2^ of > 0.8. Additionally, the SNPs were prioritized according to the following criteria *i*): known SNPs associated with metabolic traits from the literature; *ii*) SNPs within coding regions (exon); *iii*) SNPs within the promoter region (2,500 bp before the start codon); *iv*) SNPs within 3′ untranslated region (UTR) (500 bp after the stop codon); and *v*) SNPs within 100 bp before an exon-intron splicing boundaries. As result, four SNPs (rs11868035, rs2297508, rs13306741 and rs62064119) were selected from unrelated Han Chinese individuals in Beijing. DNA was isolated from EDTA-treated blood samples following standard procedures. These polymorphisms were genotyped by iPlex technology based on a MassARRAY platform in all subjects. Primers for the amplification and extension reactions were designed using the Mass Array Assay Design Version 3.1 software (Sequenom, San Diego, CA, USA), and SNP genotypes were determined according to the iPLEX protocol provided by the manufacturer. Genotyping assays were performed by laboratory personnel who were blinded to the NAFLD status of each subject. The genotyping quality was examined using a detailed QC procedure consisting of a > 95% successful call rate, duplicate calling of genotypes, internal positive control samples and HWE testing.

### Statistical analysis

Unless otherwise indicated, phenotypic quantitative data were expressed as mean ± standard deviation. To evaluate differences in clinical characteristics between cases and controls, Student’s t-test, Pearson’s Chi-squared test or the nonparametric Mann–Whitney U tests were used as appropriate. HWE was checked both in cases and controls by using the chi-squared test. In the case–control studies, each SNP was tested for association with NAFLD in a logistic regression analysis, adjusted for age, sex, marriage, education or income, smoking, tea drinking and clinical characteristics. When the genotype frequency for homozygotes of the minor allele was less than 5%, carriers (heterozygotes and homozygotes individuals) of the minor allele were grouped. *P*-values were adjusted for multiple tests.

LD between SNPs was estimated in the case–control studies using Haploview4.2 (http://www.broad.mit.edu/mpg/haploview). The Haplo.stats software package was developed using the R language and was used to estimate adjusted ORs and 95% CIs for each haplotype^38^. To assess statistical significance, we performed permutation procedures to correct the P-value of single-locus association results for multiple testing. Simulations were run 1,000 times for empirical P-values. All statistical analyses were performed using R statistical software.

## Additional Information

**How to cite this article**: Peng, X.-E. *et al*. Lack of association between *SREBF-1c* gene polymorphisms and risk of non-alcoholic fatty liver disease in a Chinese Han population. *Sci. Rep.* **6**, 32110; doi: 10.1038/srep32110 (2016).

## Supplementary Material

Supplementary Information

## Figures and Tables

**Table 1:** Clinical characteristics of NAFLD patients and control subjects.

Variables	Cases (n = 593)		Controls (n = 593)		*P* value
	%	Mean (SD)	%	Mean (SD)	
Age (years)					0.11^a^
20~	11.30		14.17		
30~	26.14		30.86		
40~	25.13		23.78		
50~	25.80		20.07		
≥60	11.64		11.13		
Sex (Male)	72.41		72.41		>0.05^a^
Income (RMB/Mon)					>0.05^a^
<1000	2.65		4.38		
1000~2000	62.22		57.4		
≥2000	35.13		38.22		
Marriage (Married)	88.23		88.34		>0.05^a^
Education					>0.05^a^
≤6 years	78.2		79.12		
≤9 years	10.27		8.95		
>9 years	11.53		11.93		
Smoking	100		97.8		0.23^a^
No-smoker	66.78		69.87		
Smoker	33.22		27.93		
Tea drinking	100		100		<0.01^a^
No-drinker	55.22		41.37		
Drinker	44.78		58.63		
AST (IU/L)		25.17 (18.93)		23.63 (13.24)	<0.01^b^
ALT (IU/L)		34.75 (25.43)		28.35 (23.52)	<0.01^b^
TC (mmol/L)		5.37 (1.04)		5.06 (1.06)	<0.01^b^
TG (mmol/L)		1.88 (1.23)		1.25 (0.65)	<0.01^b^
FPG (mmol/L)		5.71 (1.16)		5.46 (1.18)	<0.01^b^
HDL-c (mmol/L)		1.370.55)		1.60 (0.67)	<0.01^b^
LDL-c (mmol/L)		3.46 (1.13)		3.10 (1.01)	<0.01^b^
SDP (mmHG)		128.83 (13.01)		120.05 (11.90)	<0.01^b^
SBP (mmHG)		80.22 (10.31)		73.42 (9.98)	<0.01^b^
BMI (kg/m^2)^		25.49 (2.85)		22.24 (2.28)	<0.01^b^

Continuous variables ar expressed as the mean (SD); categorical variables are expressed as percentages. n: number of individuals; SD: standard deviation.

^a^Obtained by the Pearson Chi-square test.

^b^Obtained by the Mann-Whitney U test.

**Table 2:** Description of SNPs identified for *SREBF1c*.

NCBI SNP reference^a^	Chromosome position^b^	Genic location	Allele^c^	MAF			*P* value for HWE
				Controls	Cases	*P*^d^	
rs62064119	17838494	Promoter1c	C/G	0.556	0.560	0.867	>0.05
rs2297508	17812003	3′-UTRexon19c	G/C	0.120	0.130	0.456	>0.05
rs11868035	17811787	3′-UTRexon19c	A/G	0.121	0.1331	0.387	>0.05
rs13306741	17811708	3′-UTRexon19c	C/A	0.041	0.047	0.694	>0.05

HWE: Hardy-Weinberg equilibrium; MAF: minor allele frequency.

^a^Single nucleotide polymorphisms on the NCBI Reference Assembly.

^b^SNP position in the NCBI dbSNP database (http://www.ncbi.nlm.nih.gov/SNP).

^c^major/minor allele.

^d^After correcting for multiple testing by the Haploview program using 1,000 permutations.

**Table 3:** Association between the genotypes of *SREBF1C* and risk of NAFLD.

Polymorphism ID no.	Genotypes	Cases	Controls	OR and 95% CI	*P* value^a^
		n(%)	n(%)		
rs62064119					
	CC	176 (29.68)	175 (29.51)	1.00 (Reference)	—
	CG	312 (52.61)	310 (52.28)	0.94 (0.68~1.31)	0.72
	GG	105 (17.71)	108 (18.21)	0.78 (0.50~1.22)	0.62
CG + GG *vs*. CC				0.99 (0.76~1.31)	0.97
rs2297508					
	GG	448 (75.55)	460 (77.57)	1.00 (Reference)	—
	GC	136 (22.93)	124 (20.91)	1.22 (0.86~1.75)	0.26
	CC	9 (1.52)	9 (1.52)	0.60 (0.16~2.17)	0.43
GC + CC *vs*. GG				1.20 (0.89~1.62)	0.23
rs11868035	AA	446 (75.21)	458 (77.23)	1.00 (Reference)	
	AG	136 (22.93)	126 (21.25)	1.18 (0.83~1.69)	0.36
	GG	11 (1.85)	9 (1.52)	0.76 (0.23~2.55)	0.66
AG + GG *vs*. AA				1.18 (0.87~1.58)	0.29
rs13306741	GG	541 (91.23)	541 (91.23)	1.00 (Reference)	
	GT	48 (8.09)	52 (8.77)	0.98 (0.62~1.55)	0.92
	TT	4 (0.67)	0 (0.00)	—	—
GT + TT *vs*. GG				1.02 (0.65~1.60)	0.932

n: number of individuals; OR (odds ratio), determined using logistic regression and adjusted for sex, age, body mass index and other clinical characteristics; CI: confidence interval.

^a^P-value based on the Wald test.

**Table 4:** Comparison of liver function tests among the different genotypes of *SREBF1C* in different subjects.

SNP	AST			ALT		
	Cases	Controls	Total	Cases	Controls	Total
rs11868035						
AA	25.53 ± 21.16	24.52 ± 35.80	25.03 ± 29.37	36.21 ± 41.87	29.89 ± 83.40	33.06 ± 65.98
AG + GG	24.10 ± 8.95	22.66 ± 10.94	23.5 ± 10.19	30.18 ± 15.92	23.22 ± 16.03	26.99 ± 16.50^a^
rs2297508						
GG	25.57 ± 21.12	24.50 ± 35.72	25.04 ± 29.31	36.19 ± 41.77	29.87 ± 83.22	33.04 ± 65.84
CG + CC	23.94 ± 8.91	22.70 ± 11.02	23.34 ± 9.99	30.16 ± 16.02	23.21 ± 16.11	26.78 ± 16.41^a^
rs13306741						
GG	25.24 ± 19.67	24.12 ± 33.00	24.69 ± 27.09	35.09 ± 38.81	28.82 ± 76.80	31.99 ± 60.71
GT (n = 52)	24.56 ± 7.49	23.73 ± 14.68	24.14 ± 11.64	31.10 ± 14.33	23.42 ± 17.44	27.22 ± 16.35
rs62064119						
CC	23.76 ± 8.64	23.22 ± 10.90	23.49 ± 9.82	31.19 ± 19.56	24.05 ± 16.58	27.65 ± 18.47
CG + GG	25.77 ± 21.81	24.45 ± 37.18	25.12 ± 30.40	36.23 ± 42.54	30.12 ± 42.52	33.20 ± 68.17

^**a**^P < 0.05.

**Table 5:** Main haplotype frequencies of two SREBP-1cgenetic polymorphisms in NAFLD patients and controls in a Chinese population.

Haplotype^a^	NAFLD(freq.)	Control(freq.)	χ^2^	*P*^b^
AC	1042(0.879)	1024(0.866)	0.799	0.371
GG	142(0.120)	152(0.129)	0.428	0.513
GC	2(0.002)	6(0.005)	2.02	0.155

^a^In the order of rs11868035 and rs2297508.

^b^Generated by permutation test with 1000 times simulation.

^c^Global-stat = 2.49, *P* = 0.288.
