# Supplementary material for: Lack of association between SREBF-1c gene polymorphisms and risk of non-alcoholic fatty liver disease in a Chinese Han population
Source: Sci Rep. 2016 Aug 30;6:32110. doi: 10.1038/srep32110 (PMC5004200; doi:10.1038/srep32110)
Supplement: Supplementary Information [file srep32110-s1.doc]

**Lack of association between *SREBF-1c* gene polymorphisms and risk of nonalcoholic fatty liver disease in a Chinese Han population**

Xian-E Peng^a,b1^, Feng-Lin Chen ^d1^, Wenjuan Liu ^a^, ZhiJian Hu^a^, Xu Lin ^b,c*^

^a^ Department of Epidemiology and Health Statistics, the Key Laboratory of Environment and Health, universities and colleges in Fujian, Schoo l of Public Health, Fujian Medical University, 1 Xueyuan Road, Minhou, Fuzhou 350108, China

^b^ Key Laboratory of Ministry of Education for Gastrointestinal Cancer, Fujian Medical University, 1 Xueyuan Road, Minhou, Fuzhou 350108, China

^c^ Fujian Key Laboratory of Tumor Microbiology, Fujian Medical University, 1 Xueyuan Road, Minhou, Fuzhou 350108, China

^d^ Department of Gastroenterology, Union Hospital of Fujian Medical University, 29 Xinquan Road, Fuzhou 35001, China

^1^ Both authors contributed equally to this work.

^*^Corresponding author. Xu Lin, Key Laboratory of Ministry of Education for Gastrointestinal Cancer, Fujian Medical University, 1 Xueyuan Road, Minhou, Fuzhou 350108, China.

Phone: +86-591-83569986

Fax: +86-591-83569132

E-mail: linxu@mail.fjmu.edu.cn

| Quantitative | NAFLD | |  |  | Control | |  | |
| --- | --- | --- | --- | --- | --- | --- | --- | --- |
| Phenotype | AA (n=446) | AG+GG (n=147) | *P^a^* |  | AA (n=458） | AG+GG (n=135) | *P^a^* | |
| Age (years) | 46.11 ± 13.10 | 46.85 ± 12.60 | 0.56 |  | 44.09 ± 13.11 | 42.28 ± 12.56 | 0.156 | |
| BMI (kg/m^2^) | 25.41 ± 2.70 | 25.74 ± 3.28 | 0.23 |  | 22.28 ± 2.28 | 22.11 ± 2.25 | 0.44 | |
| FPG (mmol/L) | 5.71 ± 1.18 | 5.73 ± 1.08 | 0.81 |  | 5.50 ± 1.26 | 5.33 ± 0.85 | 0.14 | |
| TC (mmol/L) | 5.35 ± 0.99 | 5.44 ± 1.20 | 0.36 |  | 5.06 ± 1.00 | 5.05 ± 1.26 | 0.93 | |
| TG (mmol/L) | 1.86 ± 1.21 | 1.97 ± 1.38 | 0.33 |  | 1.26 ± 0.68 | 1.09 ± 0.45 | 0.004 | |
| HDL-C (mmol/L) | 1.36 ± 0.39 | 1.40 ± 0.87 | 0.46 |  | 1.60 ± 0.72 | 1.63 ± 0.44 | 0.55 | |
| LDL-C (mmol/L) | 3.46 ± 1.10 | 3.45 ± 1.20 | 0.92 |  | 3.12 ± 1.00 | 3.00 ± 1.05 | 0.23 | |
| SBP (mm Hg) | 128.69 ± 12.89 | 129.25 ± 13.43 | 0.65 |  | 120.31 ± 12.14 | 119.19 ± 11.07 | 0.34 | |
| DBP (mm Hg) | 80.33 ± 10.36 | 79.89 ± 10.18 | 0.66 |  | 73.78 ± 10.28 | 72.26 ± 8.89 | 0.12 | |
| AST (IU/L) | 25.53 ± 21.16 | 24.10 ± 8.95 | 0.43 |  | 24.52 ± 35.80 | 22.66 ± 10.94 | 0.55 | |
| ALT (IU/L) | 36.21 ± 41.87 | 30.18 ± 15.92 | 0.09 |  | 29.89 ± 83.40 | 23.22 ± 16.03 | 0.36 | |
| **Table S1. Comparison of various quantitative phenotypes among the different genotypes at rs11868035 in *PNPLA3* in patients with NAFLD and control subjects.**  Data are represented as the mean ± SD. *a*: *P*-values were analyzed using the Kruskal–Wallis test in each group of NAFLD and control subjects. | | | | | | | |  |

| Quantitative | NAFLD | | |  | |  | | Control | | | |  | | | |
| --- | --- | --- | --- | --- | --- | --- | --- | --- | --- | --- | --- | --- | --- | --- | --- |
| Phenotype | GG (n=448) | | CG+CC (n=145) | *P^a^* | |  | | GG (n=460) | CG+CC (n=133) | | | *P^a^* | | | |
| Age (years) | 46.16 ± 13.06 | | 46.72 ± 12.73 | 0.57 | |  | | 44.07 ± 13.09 | 42.34 ± 12.64 | | | 0.18 | | | |
| BMI (kg/m^2^) | 25.42 ± 2.69 | | 25.72 ± 3.30 | 0.31 | |  | | 22.27 ± 2.28 | 22.12 ± 2.26 | | | 0.51 | | | |
| FPG (mmol/L) | 5.70 ± 1.18 | | 5.74 ± 1.08 | 0.69 | |  | | 5.50 ± 1.26 | 5.33 ± 0.86 | | | 0.15 | | | |
| TC (mmol/L) | 5.36 ± 0.99 | | 5.43 ± 1.19 | 0.32 | |  | | 5.06 ± 1.00 | 5.06 ± 1.27 | | | 0.94 | | | |
| TG (mmol/L) | 1.87 ± 1.23 | | 1.92 ± 1.34 | 0.09 | |  | | 1.27 ± 0.69 | 1.08 ± 0.44 | | | 0.00 | | | |
| HDL-C (mmol/L) | 1.36 ± 0.39 | | 1.40 ± 0.88 | 0.51 | |  | | 1.60 ± 0.72 | 1.64 ± 0.44 | | | 0.55 | | | |
| LDL-C (mmol/L) | 3.46 ± 1.10 | | 3.45 ± 1.20 | 0.31 | |  | | 3.12 ± 1.00 | 3.01 ± 1.06 | | | 0.24 | | | |
| SBP (mm Hg) | 128.74 ± 12.86 | | 129.09 ± 13.53 | 0.77 | |  | | 120.35 ± 12.20 | 119.05 ± 10.83 | | | 0.27 | | | |
| DBP (mm Hg) | 80.36 ± 10.33 | | 79.78 ± 10.30 | 0.37 | |  | | 73.76 ± 10.31 | 72.29 ± 8.72 | | | 0.13 | | | |
| AST (IU/L) | 25.57 ± 21.12 | | 23.94 ± 8.91 | 0.93 | |  | | 24.50 ± 35.72 | 22.70 ± 11.02 | | | 0.57 | | | |
| ALT (IU/L) | 36.19 ± 41.77 | | 30.16 ± 16.02 | 0.61 | |  | | 29.87 ± 83.22 | 23.21 ± 16.11 | | | 0.36 | | | |
| **Table S2. Comparison of various quantitative phenotypes among the different genotypes at rs2297508 in patients with NAFLD and control subjects.**  Data are represented as the mean ± SD. *a*: *P*-values were analyzed using the Kruskal–Wallis test in each group of NAFLD and control subjects. | | | | | | | | | | | | | |  | |
| Quantitative | | NAFLD | |  |  | | Control | | | |  | | | |  |
| Phenotype | | GG (n=541) | GT (n=52) | *P^a^* |  | | GG (n=541) | | | GT+TT (n=52) | *P^a^* | | | |  |
| Age (years) | | 46.21 ± 12.93 | 47.22 ± 13.55 | 0.60 |  | | 43.57 ± 13.04 | | | 44.66 ± 12.65 | 0.57 | | | |  |
| BMI (kg/m^2^) | | 25.49 ± 2.86 | 25.46 ± 2.72 | 0.94 |  | | 22.21 ± 2.28 | | | 22.54 ± 2.25 | 0.31 | | | |  |
| FPG (mmol/L) | | 5.71 ± 1.19 | 5.72 ± 0.72 | 0.98 |  | | 5.47 ± 1.21 | | | 5.4 ± 0.81 | 0.69 | | | |  |
| TC (mmol/L) | | 5.36 ± 1.02 | 5.51 ± 1.25 | 0.33 |  | | 5.08 ± 1.08 | | | 4.92 ± 0.85 | 0.32 | | | |  |
| TG (mmol/L) | | 1.85 ± 1.16 | 2.30 ± 1.95 | 0.01 |  | | 1.24 ± 0.67 | | | 1.08 ± 0.42 | 0.09 | | | |  |
| HDL-C (mmol/L) | | 1.37 ± 0.56 | 1.35 ± 0.29 | 0.83 |  | | 1.6 ± 0.68 | | | 1.66 ± 0.46 | 0.51 | | | |  |
| LDL-C (mmol/L) | | 3.48 ± 1.12 | 3.23 ± 1.16 | 0.13 |  | | 3.11 ± 1.02 | | | 2.96 ± 0.95 | 0.31 | | | |  |
| SBP (mm Hg) | | 128.51 ± 12.47 | 132.20 ± 17.57 | 0.05 |  | | 120 ± 11.89 | | | 120.5 ± 12.14 | 0.77 | | | |  |
| DBP (mm Hg) | | 80.04 ± 10.18 | 82.10 ± 11.52 | 0.17 |  | | 73.54 ± 10.14 | | | 72.23 ± 8.21 | 0.37 | | | |  |
| AST (IU/L) | | 25.24 ± 19.67 | 24.56 ± 7.49 | 0.81 |  | | 24.12 ± 33.00 | | | 23.73 ± 14.68 | 0.93 | | | |  |
| ALT (IU/L) | | 35.09 ± 38.81 | 31.10 ± 14.33 | 0.47 |  | | 28.82 ± 76.80 | | | 23.42 ± 17.44 | 0.61 | | | |  |
| **Table S3. Comparison of various quantitative phenotypes among the different genotypes at rs13306741 in patients with NAFLD and control subjects.**  Data are represented as the mean ± SD. *a*: *P*-values were analyzed using the Kruskal–Wallis test in each group of NAFLD and control subjects. | | | | | | | | | | | | |  | | |

| Quantitative | NAFLD | |  |  | Control | | | |  |
| --- | --- | --- | --- | --- | --- | --- | --- | --- | --- |
| Phenotype | CC (n=176) | CG+GG (n=417) | *P^a^* |  | CC (n=175 | | CG+GG (n=418) | | *P^a^* |
| Age (years) | 46.11 ± 12.90 | 46.37 ± 13.02 | 0.83 |  | | 43.38 ± 12.50 | | 43.79 ± 13.21 | 0.73 |
| BMI (kg/m^2^) | 25.43 ± 3.21 | 25.51 ± 2.69 | 0.73 |  | | 22.23 ± 2.24 | | 22.24 ± 2.30 | 0.96 |
| FPG (mmol/L) | 5.70 ± 0.85 | 5.72 ± 1.26 | 0.86 |  | | 5.40 ± 0.68 | | 5.48 ± 1.34 | 0.46 |
| TC (mmol/L) | 5.37 ± 0.98 | 5.38 ± 1.06 | 0.89 |  | | 5.15 ± 1.08 | | 5.02 ± 1.06 | 0.19 |
| TG (mmol/L) | 1.86 ± 1.24 | 1.90 ± 1.26 | 0.72 |  | | 1.23 ± 0.62 | | 1.23 ± 0.66 | 0.9 |
| HDL-C (mmol/L) | 1.33 ± 0.30 | 1.38 ± 0.62 | 0.25 |  | | 1.58 ± 0.35 | | 1.61 ± 0.76 | 0.58 |
| LDL-C (mmol/L) | 3.40 ± 0.98 | 3.48 ± 1.18 | 0.46 |  | | 3.20 ± 1.13 | | 3.05 ± 0.96 | 0.11 |
| SBP (mm Hg) | 129.07 ± 12.12 | 128.73 ± 13.38 | 0.77 |  | | 120.41 ± 11.71 | | 119.90 ± 11.99 | 0.64 |
| DBP (mm Hg) | 79.96 ± 10.19 | 80.33 ± 10.37 | 0.69 |  | | 73.18 ± 9.36 | | 73.52 ± 10.24 | 0.71 |
| AST (IU/L) | 23.76 ± 8.64 | 25.77 ± 21.81 | 0.24 |  | | 23.22 ± 10.90 | | 24.45 ± 37.18 | 0.67 |
| ALT (IU/L) | 31.19 ± 19.56 | 36.23 ± 42.54 | 0.14 |  | | 24.05 ± 16.58 | | 30.12 ± 42.52 | 0.36 |
| **Table S4. Comparison of various quantitative phenotypes among the different genotypes at rs62064119 in patients with NAFLD and control subjects.**  Data are represented as the mean ± SD. *a*: *P*-values were analyzed using the Kruskal–Wallis test in each group of NAFLD and control subjects. | | | | | | | | | |
